# Performance across different versions of an artificial intelligence model for screen-reading of mammograms

**DOI:** 10.1007/s00330-025-12240-6

**Published:** 2026-01-13

**Authors:** Marthe Larsen, Christoph I. Lee, Marie B. Bergan, Åsne S. Holen, Håkon Lund-Hanssen, Solveig R. Hoff, Steinar Auensen, Jan F. Nygård, Kristina Lång, Yan Chen, Giske Ursin, Solveig Hofvind

**Affiliations:** 1 Department of Breast Cancer Screening, The Cancer Registry, Norwegian Institute of Public Health, Oslo, Norway; 2 Department of Radiology, University of Wisconsin-Madison School of Medicine and Public Health, Madison, WI USA; 3 Department of Radiology and Nuclear Medicine, St Olavs University Hospital, Trondheim, Norway; 4 Department of Radiology, Møre og Romsdal Hospital Trust, Ålesund, Norway; 5 Department of Health Sciences in Ålesund, Faculty of Medicine and Health Sciences, Norwegian University of Science and Technology (NTNU), Trondheim, Norway; 6 Department of Registry Informatics, The Cancer Registry, Norwegian Institute of Public Health, Oslo, Norway; 7 Diagnostic Radiology, Translational Medicine, Lund University, Lund, Sweden; 8 Unilabs: Mammography Unit, Skåne University Hospital, Malmö, Sweden; 9 School of Medicine, University of Nottingham, Clinical Science Building, Nottingham City Hospital, Nottingham, United Kingdom; 10 The Cancer Registry, Norwegian Institute of Public Health, Oslo, Norway; 11 Institute of Basic Medical Sciences, University of Oslo, Oslo, Norway; 12 Department of Preventive Medicine, University of Southern California, Los Angeles, CA USA; 13 Department of Health and Care Sciences, Faculty of Health Sciences, UiT The Arctic University of Norway, Tromsø, Norway

**Keywords:** Mammography, Screening, Artificial intelligence, Breast cancer

## Abstract

**Objectives:**

Studies have reported promising results regarding artificial intelligence (AI) as a tool for improved mammographic screening interpretive performance. We analyzed AI malignancy risk scores from two versions of the same commercial AI model.

**Materials and methods:**

This retrospective cohort study used data from 117,709 screening examinations performed in BreastScreen Norway 2009–2018. The mammograms were processed by two versions of the commercially available AI model, Transpara (version 1.7 and 2.1). The distributions of exam-level risk scores (AI score 1–10) and risk categories were evaluated for both AI versions on all examinations, including 737 screen-detected and 200 interval cancers. Scores between 1–7 were categorized as low risk, 8–9 as intermediate risk, and 10 as high risk of malignancy.

**Results:**

Area under the receiver operating curve was 0.908 (95% CI: 0.986–0.920) for version 1.7 and 0.928 (95% CI: 0.917–0.939) for 2.1 when screen-detected and interval cancers were considered as positive cases (*p* < 0.001). A total of 87.1% (642/737) and 93.5% (689/737) of the screen-detected cancers had an AI score of 10 with version 1.7 and 2.1, respectively. Among interval cancers, 45.0% (90/200) had AI score 10 with version 1.7 and 44.5% (89/200) had AI score 10 with version 2.1.

**Conclusion:**

A higher proportion of screen-detected breast cancers had the highest AI score of 10 with the newer version of the AI model compared to the older version. For interval cancers, there was no difference in the proportion of cases assigned to the highest score between the two versions.

**Key Points:**

***Question**** Studies have reported promising results regarding the use of AI in mammography screening, but comparisons of updated versus older versions are less studied*.

***Findings**** In our study, 87.1% (642/737) of the screen-detected cancers were classified with a high malignancy risk score by the old version, while it was 93.5% (689/737) for the newer version*.

***Clinical relevance**** Understanding how version updates of AI models might impact screening mammography performance will be important for future quality assurance and validation of AI models*.

**Graphical Abstract:**

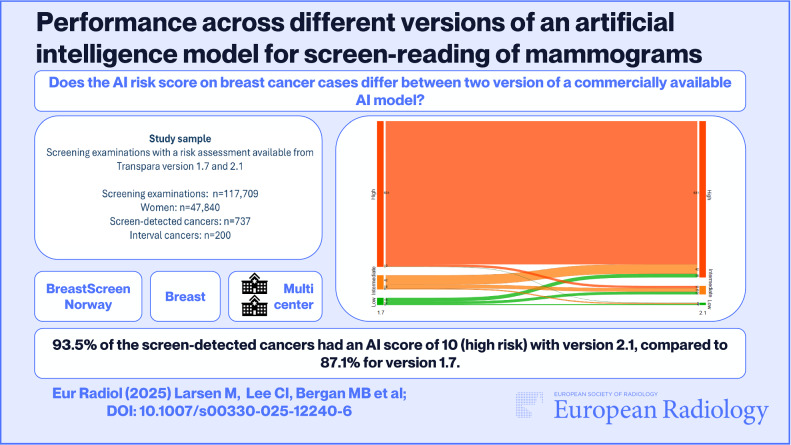

## Introduction

Breast cancer screening with mammography is expected to face major changes during the next decade [1, 2]. Artificial intelligence (AI) holds significant promise in improving mammography screening interpretation and outcomes [3–12]. Multiple Conformité Européenne (CE) marked and U.S Food and Drug Administration (FDA) cleared AI models are now available. These AI models can be used as decision support at the time of radiologists’ image interpretation, replacing a proportion of human reading in double reading settings, and/or for triaging examinations to single or double reading based on a score indicating risk of malignancy. Benefits may include an increased rate of screen-detected cancer, reduced rates of interval cancer and false positive screening results, and reduced workload for radiologists. However, there are ethical and legal challenges related to using AI, in addition to other issues such as automation bias and the cost of installation, running the algorithms, and validation and quality assurance of the performance of the systems.

Regulatory-cleared commercial AI models are undergoing improvements over time, with regular updates to the software. One study has reported results on performance for two successive versions of an AI mammography model and showed that the most recent version outperformed humans in terms of sensitivity [13]. In real-world practice, one of the major challenges of using AI in mammography interpretation is how interpretive performance may be influenced by each version update. For instance, AI thresholds for defining suspicious versus non-suspicious findings may change over time due to improvements in the model and could ultimately change screening outcomes at a population level.

Since data on differences in performance between two versions of the same commercial AI model for mammographic screening interpretation is scarce, our study objective was to evaluate how a version update impacted screening interpretive performance in a national screening program, BreastScreen Norway. In this study, we used retrospective individual, exam-level data to compare AI malignancy risk scores from two versions of an AI model in relation to key performance measures such as screen-detected and interval cancer rates. Furthermore, we compared differences in histopathological tumor characteristics and mammographic features by AI malignancy risk scores for the two versions.

## Materials and methods

This retrospective cohort study included mammograms and exam-level data on women screened in BreastScreen Norway, 2009–2018. The study was approved by the Regional Committees for Medical and Health Research Ethics (#2018/2574) and had a legal basis in accordance with Articles 6(1)(e) and 9(2)(j) of the General Data Protection Regulation. The data were disclosed on a legal basis in the Cancer Registry Regulations section 3–1 and the Personal Health Data Filing System Act section 19a to 19h [14, 15].

### Study population

BreastScreen Norway is a nationwide program and invites women aged 50–69 to biennial mammography screening, and about 250,000 women participate each year [16]. The attendance rate has been stable at about 75% since the program started in 1996 [16, 17]. Two radiologists independently interpret the mammograms and assign a score from 1 to 5 to each breast, where a score of 1 indicates normal findings, 2 probably benign findings, 3 intermediate suspicion, 4 probably malignant, and 5 high suspicion of malignancy. Examinations with a score of 2 or higher by either or both radiologists are discussed in a consensus meeting, where it is decided if a woman should be recalled for further assessment or not.

**Fig. 1:**
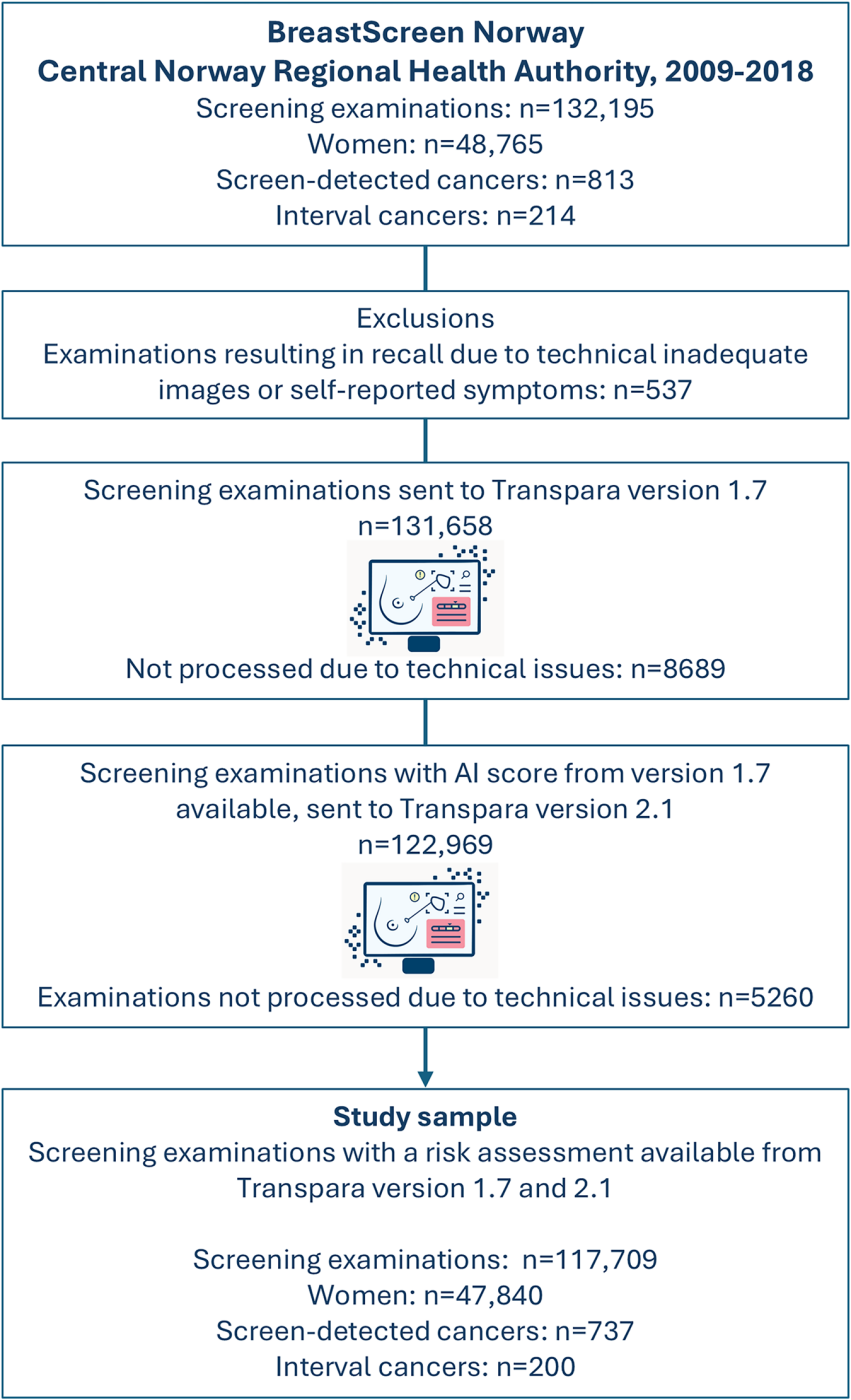
Flowchart of the study population

The overall study population included information on 132,195 standard 2D digital screening examinations performed at two breast centers in the Central Norway Regional Health Authority between January 2009 and December 2018 (Fig. 1). After exclusions, the final sample included 117,709 screening examinations. At these two centers, all mammograms were acquired with Siemens Mammomat INSPIRATION during the study period. We have previously reported the performance of version 1.7 for a similar sample of examinations [18, 19].

### AI model

Transpara versions 1.7 and 2.1 (ScreenPoint Medical BV) were used to score each of the mammography examinations. The newest version was available for clinical use beginning in December 2024 [20] and is FDA-cleared and CE-marked for both 2D and 3D mammography [20, 21]. Version 2.1 comes with changes in the architectural algorithm, and according to the AI company, more data from different mammography vendors and breast centers have been included in the training of the model, in addition to updated data sampling techniques. We used an exam-level risk score (AI score) categorization, where AI scores 1–7 indicate low risk of breast cancer, 8–9 intermediate risk, and 10 high malignancy risk. The model also provides an exam-level continuous risk score.

### Definitions of variables

Screen-detected cancer was defined as invasive breast cancer or ductal carcinoma in situ (DCIS) diagnosed after a recall assessment. A recall was defined as an assessment due to suspicious findings on the screening mammograms, while a false positive screening result was defined as a recall assessment without any breast cancer detected at the follow-up assessment. Interval cancers were defined as invasive cancer or DCIS diagnosed within 24 months after a negative screening examination or 6–24 months after a false positive screening result. We included only screening examinations taken prior to a diagnosis of interval cancer in our study.

Histopathological type was classified as DCIS or invasive cancer. For invasive cancers, we analyzed median tumor diameter (mm), histological grade (1–3), lymph node status (positive/negative), and molecular subtypes based on immunohistochemistry. Subtypes were classified according to a slightly modified St. Gallen definition: Luminal A (ER-positive, HER2-negative, Ki-67 ≤ 30% or grade 1 or 2 if Ki-67 is missing), luminal B HER2-negative (ER-positive, HER2-negative, Ki-67 > 30% or grade 3 if Ki-67 is missing), luminal B HER2-positive (ER-positive, HER2-positive), HER2-positive (ER-negative, PR-negative, and HER2-positive), and triple negative (ER-negative, PR-negative, and HER2-negative) [22]. Mammographic features were assessed by radiologists according to a modified BI-RADS classification for recalled cases in the standard screening setting and were categorized as “mass,” “spiculated mass,” “architectural distortion,” “asymmetric density,” “density with calcifications,” and “calcifications alone” [16, 23].

### Statistical analysis

The area under the receiver operating curve (AUC) and 95% confidence intervals (CI) were estimated for the continuous AI score, with one AUC estimate for screen-detected cancers as true positives and another using cancers detected within 2 years (screen-detected and interval cancers). Cluster bootstrap analysis was performed to account for several attendances for each woman. Differences between the AUC estimates were tested using the *roccomp* command in stata, and *p*-values < 0.05 were considered statistically significant [24]. Stratified by the two AI versions, the distribution of AI score 1–10 was described in terms of consensus, dismissed at consensus, recall, false positives, screen-detected cancers and interval cancers, as well as for all examinations.

Histopathological tumor characteristics and mammographic features were described for invasive cancers stratified by different AI score groups. Mammographic features were not described for interval cancers due to a limited number of cases with available information in each AI score group. The AI groups were (1) increased score (AI score 1–9 with 1.7 and 10 with 2.1), (2) stable low or intermediate score (AI score 1–9 with both versions), (3) stable high score (AI score 10 with both versions), and (4) decreased score (AI score 10 with 1.7 and 1–9 with 2.1).

We used Stata (StataCorp. 2023. Stata Statistical Software: Release 19) to analyze the data.

## Results

After excluding examinations resulting in a recall due to technically inadequate images or self-reported symptoms, and examinations where AI risk assessment was missing from the overall study population, the final study sample included 117,709 screening examinations, including 737 screen-detected and 200 interval cancers (Fig. 1). The examinations were performed among 47,840 women. The mean age at screening was 59.9 years (standard deviation, SD = 5.9), and the mean age at diagnosis was 61.2 years (SD = 5.8 years).

### Accuracy based on the continuous AI risk score

When screen-detected cancers (*n* = 737) were considered as positive cases, AUC was 0.949 (95% CI: 0.939–0.959) for version 1.7 and 0.976 (95% CI: 0.970–0.981) for version 2.1, *p* < 0.001 (Table 1). The values were 0.908 (95% CI: 0.986–0.920) for 1.7 and 0.928 (95% CI: 0.917–0.938) for 2.1 when both screen-detected and interval cancers (*n* = 937) were considered positive cases, *p* < 0.001.

**Table 1:** Area under the receiver operating curve (AUC) with 95% confidence interval (CI) for version 1.7 and 2.1 of Transpara when considering screen-detected (*n* = 737) and screen-detected + interval cancers (*n* = 937) among 117,709 screening examinations as true positives

Version of Transpara	Screen-detected cancer AUC (95% CI)	Screen-detected and interval cancer AUC (95% CI)
1.7	0.949 (0.939–0.959)	0.908 (0.896–0.920)
2.1	0.976 (0.970–0.981)	0.928 (0.917–0.938)
*p*-value	< 0.001	< 0.001

### AI score 1–10

Version 1.7 assigned an AI score of 1 to 12.2% (14,347/117,709) of examinations, while version 2.1 assigned a score of 1 to 9.0% (10,563/117,709) of examinations (Table 2). An AI score of 10 was assigned to 10.1% (11,963/117,709) of examinations for version 1.7 and 10.7% (12,605/117,709) for version 2.1. The negative predictive value for low AI scores (examination not registered with screen-detected or interval cancer/AI score 1–7) was 99.9% for both version 1.7 and 2.1 (80,043/80,136 for 1.7 and 80,406/80,483 for 2.1).

**Table 2:** Distribution of artificial intelligence (AI) score 1–10 for Transpara versions 1.7 and 2.1 for all examinations, screen-detected cancers, interval cancers and false-positive screening results

	All examinations		Screen-detected cancers		Interval cancers		False positives	
AI score	1.7	2.1	1.7	2.1	1.7	2.1	1.7	2.1
1	14,347 (12.2%)	10,563 (9.0%)	5 (0.7%)	0 (-)	9 (4.5%)	2 (1.0%)	49 (1.6%)	31 (1.0%)
2	5930 (5.0%)	10,684 (9.1%)	0 (-)	1 (0.1%)	1 (0.5%)	14 (7.0%)	67 (2.2%)	49 (1.6%)
3	13,166 (11.2%)	11,074 (9.4%)	0 (-)	0 (-)	9 (4.5%)	6 (3.0%)	136 (4.4%)	98 (3.2%)
4	12,286 (10.4%)	11,173 (9.5%)	8 (1.1%)	2 (0.3%)	9 (4.5%)	5 (2.5%)	187 (6.1%)	130 (4.2%)
5	11,960 (10.2%)	13,023 (11.1%)	4 (0.5%)	1 (0.1%)	11 (5.5%)	8 (4.0%)	212 (6.9%)	220 (7.2%)
6	11,082 (9.4%)	11,750 (10.0%)	9 (1.2%)	2 (0.3%)	9 (4.5%)	11 (5.5%)	281 (9.2%)	237 (7.7%)
7	11,355 (9.7%)	12,216 (10.4%)	6 (0.8%)	4 (0.5%)	13 (6.5%)	21 (10.5%)	327 (10.7%)	327 (10.7%)
8	12,303 (10.5%)	13,075 (11.1%)	18 (2.4%)	10 (1.4%)	14 (7.0%)	17 (8.5%)	436 (14.2%)	461 (15.0%)
9	13,334 (11.3%)	11,546 (9.8%)	45 (6.0%)	28 (3.8%)	35 (17.5%)	27 (13.5%)	602 (19.6%)	555 (18.1%)
10	11,963 (10.1%)	12,605 (10.7%)	642 (87.1%)	689 (93.5%)	90 (45.0%)	89 (44.5%)	774 (25.2%)	963 (31.4%)
Total	117,709 (100%)	117,709 (100%)	737 (100%)	737 (100%)	200 (100%)	200 (100%)	3071 (100%)	3071 (100%)

We found that 87.1% (642/737) of the screen-detected cancers were assigned an AI score of 10 with version 1.7 compared to 93.5% (689/737) with 2.1 (Table 2). The proportion of screen-detected cancers among all examinations with an AI score of 10 was 5.4% (642/11,963) for 1.7 and 5.5% (689/12,605) for 2.1. For interval cancers, the percentage with AI score 10 was 45.0% (90/200) and 44.5% (89/200) for version 1.7. and 2.1, respectively.

In the standard double reading setting, 3071 false positive screening results were registered (Table 2). For version 1.7, 25.2% (774/3071) of these were assigned an AI score of 10 compared to 31.4% (963/3071) for version 2.1. The distribution of screening examinations by AI score for cases discussed at consensus, dismissed at consensus and recalled is shown in Supplementary Table 1.

### AI scores in version 1.7 and 2.1 for screen-detected cancers

**Fig. 2:**
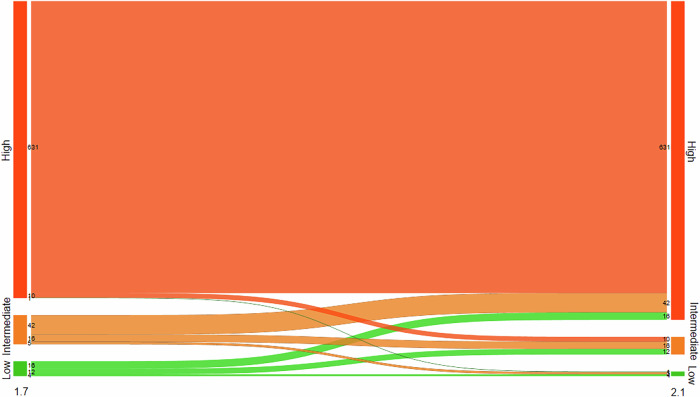
Number of screen-detected cancers with low artificial intelligence (AI) score (AI score 1–7), intermediate (AI score 8–9) and high (AI score 10) AI score using Transpara versions 1.7 and 2.1

**Fig. 3:**
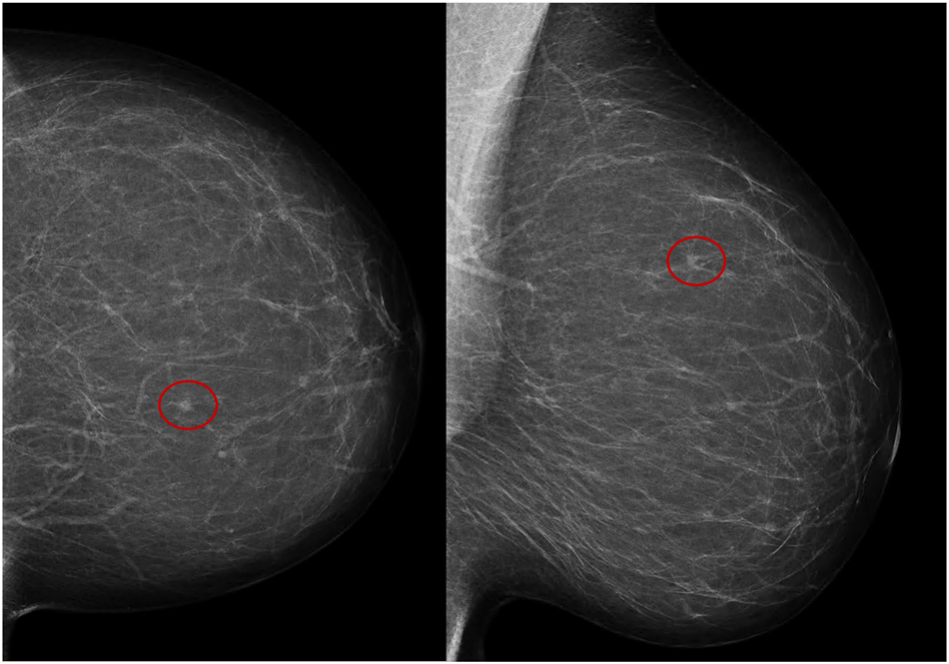
Left craniocaudal and mediolateral oblique mammograms in a 53-year-old woman with an invasive four-millimeter histologic grade 1 screen-detected cancer. The exam-level artificial intelligence (AI) score for version 1.7 was 5 and 10 for version 2.1 (increased AI score). The red circles mark the location of the tumor

Among the 737 screen-detected cancers, 7.9% (58/737) showed an increase in AI score from low or intermediate (AI score 1–9 in version 1.7) to high (AI score 10 in version 2.1) (Table 3, Fig. 2). The AI scores remained stable at a low or intermediate level for 5.0% (37/737), stable at the highest AI score for 85.6% (631/737), while 1.5% (11/737) of the cancer cases had a decreased AI score (AI score of 10 with 1.7 and 1–9 in 2.1). For the 58 cases with an increased score, 16 had an AI score of 1–7 (low risk), and 42 had a score of 9 (intermediate risk) when analyzed with version 1.7 (Fig. 2). Figure 3 shows an example of a screen-detected cancer with an increased AI score (AI score 5 with version 1.7 and 10 with version 2.1). A total of 39.7% (23/58) had discordant interpretations (an interpretation score of 1 by one radiologist and 2 or higher by the other). Among the 11 screen-detected cancers with decreased AI score with version 2.1 compared to 1.7, 3 cases had an interpretation score of 3 or higher by both radiologists, indicating intermediate to high suspicion of malignancy by both radiologists.

**Table 3:** Numbers of screen-detected cancers and invasive screen-detected cancers, and histopathological tumor characteristics and mammographic features of invasive screen-detected cancers for the different artificial intelligence (AI) score groups

	Increased score (low/intermediate to high)	Stable low/intermediate score	Stable high score	Decreased score (high to low/intermediate)	Total
AI scores (version)	1–9 (1.7) + 10 (2.1)	1–9 (1.7) + 1–9 (2.1)	10 (1.7) + 10 (2.1)	10 (1.7) + 1–9 (2.1)	1–10 (1.7) + 1–10 (2.1)
Number of cases, *n* (%*)	58 (7.9%)	37 (5.0%)	631 (85.6%)	11 (1.5%)	737 (100%)
Invasive cancers, *n* (%*)	48 (82.8%)	33 (89.2%)	526 (83.4%)	10 (90.9%)	617 (83.7%)
Histopathological characteristics of invasive cancers					
Tumor diameter, mm median (IQR)	9 (7–15)	10 (7–17)	13 (9–18)	11 (7–13)	12 (9–18)
Data not available** (*n*)	3	3	29	0	35
Histologic grade, *n* (%*)					
Grade 1	23 (51.1%)	9 (32.1%)	171 (34.3%)	2 (20.0%)	205 (35.2%)
Grade 2	13 (28.9%)	13 (46.4%)	210 (42.1%)	2 (20.0%)	238 (40.9%)
Grade 3	9 (20.0%)	6 (21.4%)	118 (23.7%)	6 (60.0%)	139 (23.7%)
Data not available** (*n*)	3	5	27	0	35
Lymph node involvement, *n* (%*)	7 (14.9%)	6 (19.4%)	117 (22.8%)	1 (10.0%)	131 (22.8%)
Data not available**, *n*	1	2	12	0	15
Immunohistochemical subtypes, *n* (%*)					
Luminal A	35 (74.5%)	22 (75.9%)	355 (68.8%)	4 (40.0%)	416 (69.1%)
Luminal B HER2-negative	5 (10.6%)	4 (13.8%)	79 (15.3%)	3 (30.0%)	91 (15.1%)
Luminal B HER2-positive	2 (4.3%)	0 (-)	33 (6.4%)	2 (20.0%)	37 (6.2%)
HER2-positive	0 (-)	0 (-)	28 (5.4%)	0 (-)	28 (4.7%)
Triple negative	5 (10.6%)	3 (10.3%)	21 (4.1%)	1 (10.0%)	30 (5.0%)
Data not available**, *n*	1	4	10	0	15
Mammographic features of invasive cancers, *n* (%*)					
Mass	14 (34.6%)	10 (37.0%)	67 (13.7%)	4 (44.4%)	95 (16.7%)
Spiculated mass	16 (37.2%)	9 (33.3%)	217 (44.4%)	3 (33.3%)	245 (43.1%)
Distortion	2 (4.7%)	3 (11.1%)	23 (4.7%)	0 (-)	28 (4.9%)
Asymmetry	11 (25.6%)	4 (14.8%)	80 (16.4%)	1 (11.1%)	96 (16.9%)
Mass with calcifications	0 (-)	0 (-)	47 (9.6%)	0 (-)	47 (8.3%)
Calcifications alone	0 (-)	1 (3.7%)	55 (11.3%)	1 (11.1%)	57 (10.0%)
Data not available**	5	6	37	1	49

* Percentages in each AI score group were calculated based on all screen-detected cancers. The percentage of invasive cases was calculated based on the number of screen-detected cancers in each group. Percentages for histologic grade 1–3, lymph node positives, subtypes, and mammographic features were calculated based on the number of invasive screen-detected cancers with available data

** Data not available due to neoadjuvant treatment or other reasons for tumor diameter and histologic grade, and data not available for other reasons for subtypes and mammographic features

Tumor diameter stratified by AI score groups showed a median tumor diameter for invasive cases of 9 mm (interquartile range, IQR: 7–15) for cases with increased score, 10 mm (IQR: 7–17) for those with stable low or intermediate score, 13 mm (IQR: 9–18) for those with stable high scores, and 11 mm (IQR: 7–13) for cases with decreased score (Table 3). Positive lymph node status was registered for 14.9% (7/47) of the invasive cases with increased score, 19.4% (6/31) of the cases with stable low or intermediate score, 22.8% (117/514) of the cases with stable high score, and 10.0% (1/10) for those with decreased score.

“Spiculated mass” was the most prevalent mammographic feature, 37.2% (16/43), among cases with increased scores (Table 3). No cases with “calcifications alone” had an increased score, but 3.7% (1/27) of the cases with stable low or intermediate score, 11.3% (55/489) of those with stable high score, and 11.1% (1/9) of those with decreased score were classified as “calcifications alone.” A total of 7 of the 11 screen-detected cancers with decreased score were classified as “mass” or “spiculated mass.”

### AI score in version 1.7 and 2.1 for the interval cancers

**Fig. 4:**
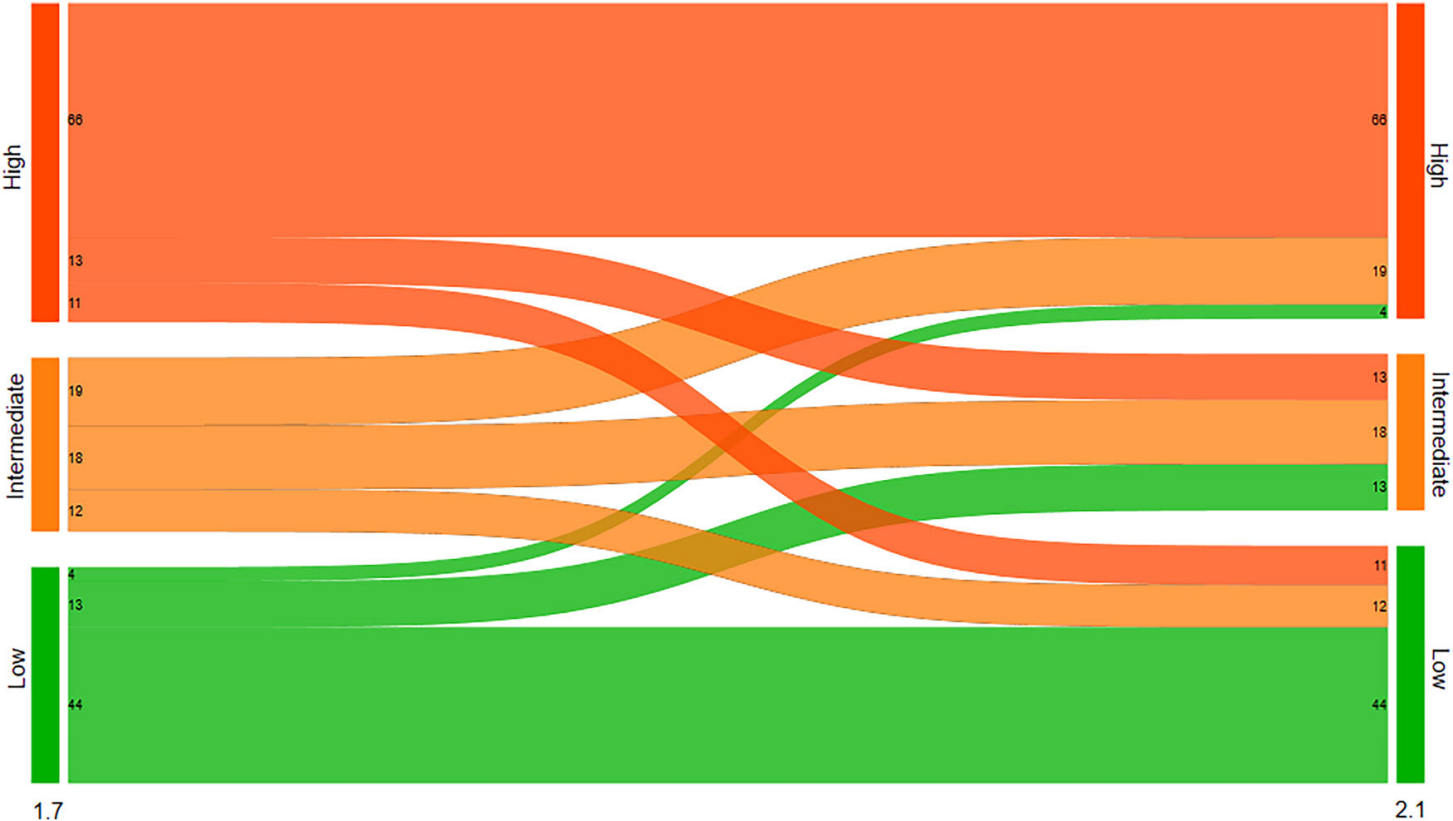
Number of interval cancers with low artificial intelligence (AI) score (AI score 1–7), intermediate (AI score 8–9) and high (AI score 10) AI score using Transpara versions 1.7 and 2.1

A total of 11.5% (23/200) of the interval cancers had an increased score, 43.5% (87/200) had stable low or intermediate score, 33.0% (66/200) had stable high score, and 12.0% (24/200) had a decreased score in version 2.1 compared to 1.7 (Table 4, Fig. 4). A total of 83.3% (20/24) of the interval cancers with decreased AI score had an interpretation score of 1 (negative) by both radiologists. Among the 89 interval cancers assigned an AI score of 10 using version 2.1, 4.5% (4/89), 21.3% (19/89) and 74.2% (66/89) were assigned an AI score of 1–7, 8–9 and 10 by version 1.7, respectively (Fig. 4).

**Table 4:** Numbers of interval cancers, invasive interval cancers, and histopathological tumor characteristics of invasive interval cancers for the different artificial intelligence (AI) score groups

	Increased score (low/intermediate to high)	Stable low/intermediate score	Stable high score	Decreased score (high to low/intermediate)	Total
AI scores (version)	1–9 (1.7) + 10 (2.1)	1–9 (1.7) + 1–9 (2.1)	10 (1.7) + 10 (2.1)	10 (1.7) + 1–9 (2.1)	1–10 (1.7) + 1–10 (2.1)
Number of cases, *n* (%*)	23 (11.5%)	87 (43.5%)	66 (33.0%)	24 (12.0%)	200 (100%)
Invasive cancers, *n* (%*)	22 (95.7%)	82 (94.3%)	62 (93.9%)	23 (95.8%)	189 (94.5%)
Histopathological characteristics of invasive cancers					
Tumor diameter, mm median (IQR)	15 (10–25)	16 (11–25)	17 (11–27)	17 (13–30)	16 (11–25)
Data not available** (*n*)	2	3	2	0	7
Histologic grade, *n* (%*)					
Grade 1	4 (20.0%)	9 (11.5%)	15 (24.6%)	4 (17.4%)	32 (17.4%)
Grade 2	8 (40.0%)	29 (36.3%)	27 (44.3%)	9 (39.1%)	73 (39.7%)
Grade 3	8 (40.0%)	42 (52.5%)	19 (31.2%)	10 (43.5%)	79 (42.9%)
Data not available** (*n*)	2	2	1	0	5
Lymph node involvement, *n* (%*)	8 (40.0%)	29 (36.3%)	19 (32.2%)	7 (31.8%)	63 (34.8%)
Data not available** (*n*)	2	2	3	1	8
Immunohistochemical subtypes, *n* (%*)					
Luminal A	14 (73.7%)	28 (34.6%)	40 (65.6%)	10 (43.5%)	92 (50.0%)
Luminal B HER2-negative	1 (5.3%)	22 (27.2%)	10 (16.4%)	3 (13.0%)	36 (19.6%)
Luminal B HER2-positive	3 (15.8%)	10 (12.4%)	3 (4.9%)	4 (17.4%)	20 (10.9%)
HER2-positive	0 (-)	10 (12.4%)	3 (4.9%)	2 (8.7%)	15 (8.2%)
Triple negative	1 (5.3%)	11 (13.6%)	5 (8.2%)	4 (17.4%)	21 (11.4%)
Data not available** (*n*)	3	1	1	0	5

* Percentages in each AI score group were calculated based on all interval cancers. The percentage of invasive cases was calculated based on the number of interval cancers in each group. Percentages for histologic grade 1–3, lymph node positives, and subtypes were calculated based on the number of invasive interval cancers with available data

** Data not available due to neoadjuvant treatment or other reasons for tumor diameter and histologic grade, and data not available for other reasons for subtypes

Among the invasive interval cancers, histologic grade 3 was observed for 40.0% (8/20), 52.5% (42/80), 31.2% (19/61), and 43.5% (10/23) of the cases with increased score, stable low or intermediate score, stable high score, and decreased score, respectively (Table 4). The corresponding numbers of cases with lymph node involvement in the four different groups were 40.0% (8/20), 36.3% (29/80), 32.2% (19/59), and 31.8% (7/22).

## Discussion

In this retrospective cohort study, we found a substantially higher proportion of screen-detected cancers assigned to the highest AI score of 10 using the newer versus older version of an FDA-cleared, CE-marked AI model (93.5% versus 87.1%, respectively). For interval cancers, no difference in the overall proportion of cases assigned to the highest score of 10 between the two versions was observed (44.5% for the newest version, 2.1, versus 45.0% for the older, 1.7).

The AUC for version 2.1 was in line with reported AUC values using other AI systems on data from BreastScreen Norway [4, 12, 25]. When including screen-detected and interval cancers as true positives, AUC was 0.928 in this study, compared to 0.928 and 0.921 using two other CE-marked AI models [4, 12]. An in-house model trained on data from BreastScreen Norway has also reported an AUC of 0.925 [25]. The similarities in AUC values may indicate that there is a maximum plateau of what we can expect for standalone accuracy of AI, and the remaining challenge may be to identify how we can best combine AI and radiologists in the interpretation of mammograms in real-world screening settings.

The randomized controlled MASAI trial from Malmø used Transpara version 1.7 as decision support in the screen-reading, and they found a 29% higher screen-detected cancer rate with single reading of examinations with AI score 1–9 and double reading of examinations with AI score 10 versus standard double reading [9]. Using the updated version of this algorithm may result in an even higher screen-detected cancer rate than reported in the MASAI trial. Interval cancer rate is the primary endpoint of the MASAI trial, and how a higher screen-detected cancer rate affects the interval cancer rate is yet to be published.

Considering the results on histopathological tumor characteristics and mammographic features, there is no obvious explanation for why more screen-detected cancer cases were assigned a score of 10 with version 2.1. Asymmetry was observed for 25.6% in the group with an increased score and 14.8% for those with a stable low or stable intermediate score. Thus, identification of suspicious asymmetries may be improved with the new version of this AI model. Despite the few cases with decreased scores in this population-based screening cohort, it is surprising that we observed screen-detected cancers with lower AI scores for an updated version. Further, the screen-detected cancer cases not classified with AI score 10 with either version (increased, decreased or stable low/intermediate) would be of great interest to analyze updated versions in the future in relation to performance drift.

Prior studies have shown that about 20–30% of interval cancers are defined as false negatives and have the potential to be detected earlier [26, 27]. In this study, we did not observe an increase in the proportion of interval cancers classified with the highest AI score, and 12% of interval cancers had lower scores for version 2.1. versus 1.7. The results suggest that there might be a limit in terms of AI-detectable visible findings, with a maximum potential for identifying interval cancers in the range of 33.0% (stable high) to 44.5% (percentage with AI score 10 with version 2.1).

Prior studies have also indicated more histopathological favorable tumor characteristics for interval cancers classified as missed/minimal signs at screening [26]. We did not identify a clear trend of favorable or non-favorable tumor characteristics between the AI score groups for interval cancers. But considering the high proportion of histological grade 3 tumors and triple negative cancers, the cases with stable low or intermediate AI score may represent true interval cancers, where we would not expect high AI scores due to the lack of suspicious mammography findings on the screening images.

Strengths of our study include the large screening cohort with complete capture of cancer outcomes. Limitations include the use of mammography examinations acquired from a single manufacturer (Siemens) and the use of one AI vendor. The differences in AI scores between the versions may not be transferable to other manufacturers, breast centers, and commercial AI models. We did not examine the location of AI markings on the mammography images, limiting our ability to know if any mammographic findings contribute to score changes or not. However, we reported that the AI model used in this study marked the correct cancer location for all screen-detected cancers with the highest AI score of 10 in a previous study [28].

In conclusion, we observed a higher proportion of screen-detected cancers categorized with the highest AI score when using the updated version of a regulatory-cleared commercial AI model for screening mammography interpretation. However, the net benefit of using AI in clinical practice remains unknown, as we also observed a higher proportion of cases with AI score 10, and the resulting recall and false positive screening rate remains unknown. Moreover, there was no change in the proportion of interval cancers categorized with the highest AI score with the updated version. Moving forward, understanding how commercial mammography AI model updates impact population-level screening mammography performance and outcomes will be important for future quality assurance and validation of AI models.

## Supplementary information

ELECTRONIC SUPPLEMENTARY MATERIAL

## Abbreviations

AI: Artificial intelligence
AUC: Area under the receiver operating curve
CE: Conformité Européenne
CI: Confidence interval
DCIS: Ductal carcinoma in situ
ER: Estrogen receptor
FDA: U.S Food and Drug Administration
Her2: Human epidermal growth factor receptor 2
IQR: Interquartile range
