# Supplementary material for: Performance across different versions of an artificial intelligence model for screen-reading of mammograms
Source: Eur Radiol. 2026 Jan 13;36(6):4422–33. doi: 10.1007/s00330-025-12240-6 (PMC13212409; doi:10.1007/s00330-025-12240-6)
Supplement: Supplementary file 1 — ELECTRONIC SUPPLEMENTARY MATERIAL [file 330_2025_12240_MOESM1_ESM.pdf]

Performance across different versions of an artificial intelligence model for screen-reading of mammograms

ELECTRONIC SUPPLEMENTARY MATERIAL

Supplementary Table 1. Distribution of artificial intelligence (AI) score 1-10 for cases discussed at consensus; cases dismissed at consensus and recalled cases for Transpara version 1.7 and 2.1.

	Consensus		Dismissed at consensus		Recalled	
AI score	1.7	2.1	1.7	2.1	1.7	2.1
1	347 (3.3%)	204 (2.0%)	293 (4.4%)	173 (2.6%)	54 (1.4%)	31 (0.8%)
2	256 (2.5%)	310 (3.0%)	189 (2.8%)	260 (3.9%)	67 (1.8%)	50 (1.3%)
3	577 (5.5%)	444 (4.3%)	441 (6.6%)	346 (5.2%)	136 (3.6%)	98 (2.6%)
4	737 (7.1%)	556 (5.3%)	542 (8.2%)	424 (6.4%)	195 (5.1%)	132 (3.5%)
5	812 (7.8%)	821 (7.9%)	596 (9.0%)	600 (9.0%)	216 (5.7%)	221 (5.8%)
6	927 (8.9%)	907 (8.5%)	638 (9.6%)	668 (10.0%)	290 (7.6%)	239 (6.3%)
7	1074 (10.3%)	1085 (10.4%)	741 (11.1%)	754 (11.3%)	333 (8.7%)	331 (8.7%)
8	1279 (12.2%)	1426 (13.6%)	825 (12.4%)	956 (14.4%)	454 (11.9%)	471 (12.4%)
9	1711 (16.4%)	1593 (15.2%)	1065 (16.0%)	1010 (15.2%)	647 (17.0%)	583 (15.3%)
10	2737 (26.2%)	3111 (29.8%)	1323 (19.9%)	1462 (22.0%)	1416 (37.2%)	1652 (43.4%)
Total	10 457 (100%)	10 457 (100%)	6653 (100%)	6653 (100%)	3808 (100%)	3808 (100%)
